# Cataloguing functionally relevant polymorphisms in gene DNA ligase I: a computational approach

**DOI:** 10.1007/s13205-011-0006-8

**Published:** 2011-04-27

**Authors:** Abhishek A. Singh, Dakshinamurthy Sivakumar, Pallavi Somvanshi

**Affiliations:** 1 Department of Bioinformatics-BiGCaT, Maastricht University, Maastricht, The Netherlands; 2 Department of Bioinformatics, School of Chemical and Biotechnology, SASTRA University, Thanjavur, Tamil Nadu India; 3 Bioinformatics Centre, Biotech Park, Sector-G, Jankipuram, Lucknow, Uttar Pradesh India

**Keywords:** Ligase, Mutant, SNP

## Abstract

A computational approach for identifying functionally relevant SNPs in gene LIG1 has been proposed. LIG1 is a crucial gene which is involved in excision repair pathways and mutations in this gene may lead to increase sensitivity towards DNA damaging agents. A total of 792 SNPs were reported to be associated with gene LIG1 in dbSNP. Different web server namely SIFT, PolyPhen, CUPSAT, FASTSNP, MAPPER and dbSMR were used to identify potentially functional SNPs in gene LIG1. SIFT, PolyPhen and CUPSAT servers predicted eleven nsSNPs to be intolerant, thirteen nsSNP to be damaging and two nsSNPs have the potential to destabilize protein structure. The nsSNP rs11666150 was predicted to be damaging by all three servers and its mutant structure showed significant increase in overall energy. FASTSNP predicted twenty SNPs to be present in splicing modifier binding sites while rSNP module from MAPPER server predicted nine SNPs to influence the binding of transcription factors. The results from the study may provide vital clues in establishing affect of polymorphism on phenotype and in elucidating drug response.

## Introduction

Single nucleotide polymorphisms, often referred as SNP, are the most common DNA variations present throughout human genome with a frequency of one in thousand base pairs (Brookes 1999). SNPs present in coding region are either synonymous SNP (sSNP) in which any alteration in the codon does not result in coding of different amino acid or nonsynonymous SNP (nsSNP) where a change in codon results in coding of different amino acid. The missense mutations (a category of nsSNP) are of importance because of their ability to influence protein functions and many of them are linked to human inheritable diseases (krawczak et al. 2000; Tokuriki et al. 2008; Wang and Moult 2001). While SNPs present in other genomic regions, viz untranslated regions (UTR), intron and promoter regions have potential to influence gene regulation (Mooney 2005). SNPs in transcription factor binding site (TFBS) may disrupt the site (Boccia et al. 1996; Vasiliev et al. 1999) or may form a novel binding site (Knight et al. 1999; Piedrafita et al. 1996). Similarly, a SNP in micro RNA binding site may lead to repression of protein coding genes or activators of RNA degradation (Mishra et al. 2008). Furthermore, SNPs in splicing modifiers binding site (enhancers or silencers) may generate an unstable mRNA resulting in a defective or truncated protein (ElSharawy et al. 2006). Some SNPs are functional (Hardison 2003) and thus, their identification is crucial to understand molecular basis of complex traits and diseases in human (Shastry 2002).

The experimental techniques are most comprehensive and precise ones in distinguishing functional SNPs from neutral ones (Chen and Sullivan 2003). It is not feasible in terms of time and cost to perform laboratory experiments for all SNPs in human genome (or in single gene) and elucidate their functional importance while theoretical or computational methods aid in narrowing down the number of potentially functional SNPs present in a human gene (Ramensky et al. 2002). In this study, the authors have applied web-based computational tools to identify potentially functional SNPs influencing protein stability, binding of splicing modifiers, binding of transcription factors and binding of micro RNA in gene DNA Ligase I (LIG1, ATP-dependent). The two most important processes in which gene LIG1 participates are joining of Okazaki fragments during eukaryotic DNA replication and ligation of synthesized patch during base excision repair (BER) (Pascal et al. 2004; Vago et al. 2009; Goetz et al. 2005; Lee et al. 2008; Timson et al. 2000). DNA replication gene LIG1 also interacts with proliferation cell nuclear antigen (PCNA) (Levin et al. 1997; Montecucco et al. 1998; Liang et al. 2008) and loss in its ability to interact with PCNA jeopardises its normal functionality to join Okazaki fragments and to ligate synthesized patch during BER (Liang et al. 2008; Levin et al. 2000). SNPs in gene LIG1 may cause DNA Ligase I deficiency which results in immunodeficiency and increased sensitivity to DNA-damaging agents (Barnes et al. 1992). In this study, mutant protein structures were modelled and compared with native structure of gene product LIG1, for changes in energy and Root Mean Square Deviation (RMSD) values.

The present *in silico* study focuses on identification of functional SNPs in most of genomic regions of human gene LIG1 as compared to the recent *in silico* studies which were more focussed on identification of deleterious nsSNPs (Doss et al. 2008a, b; Rajasekaran and Sethumadhavan 2010; Kanthappan and Sethumadhavan 2010).

## Materials and methods

### Dataset

The single nucleotide polymorphism database (dbSNP) (Sherry et al. 2001) cited at http://www.ncbi.nlm.nih.gov/SNP was used to retrieve SNPs and their related protein sequences for the gene LIG1.

### Identification of deleterious nonsynonymous single nucleotide polymorphism by sequence homology based method

Sorting Intolerant from Tolerant (SIFT) tool accessible at http://sift.jcvi.org/ was applied to detect deleterious nonsynonymous SNPs (Ng and Henikoff 2001, 2002, 2003; Kumar et al. 2009). SIFT compiles a dataset of functionally linked protein sequences by searching protein database using PSI-BLAST algorithm. Then, it builds an alignment from the homologous sequences with the query sequence and scans all positions in the alignment and calculates the probabilities for amino acids at that position. The substitution at each position with normalized probabilities less than a tolerance index or SIFT score of 0.05 are predicted to be deleterious or intolerant while those equivalent or greater than 0.05 are predicted to be tolerant (Ng and Henikoff 2001). In this study RefSeq ID or GI number and substitution(s) was given as input to SIFT blink program (Kumar et al. 2009). The program was executed on default settings i.e., best BLAST hits for each organism were included and sequences greater than 90% identity to query were removed. A total of thirty-one nsSNPs in protein transcript (NP_000225.1) of gene LIG1 (NM_000234.1) were analysed for identification of deleterious variant(s).

### Identification of damaging nonsynonymous single nucleotide polymorphism by structural-homology based method

Polymorphism Phenotyping tool (PolyPhen) available at http://coot.embl.de/PolyPhen/ uses structural and evolutionary characteristics to identify deleterious nsSNPs (Sunyaev et al. 2000; Ramensky et al. 2002). PolyPhen uses either amino acid sequence or SWall protein database ID (SPTR) or accession number with the two amino acid variants along with their position as inputs. The algorithm performs sequence-based characterization of the mutation site using a blend of various algorithms, followed by the identification and alignment of homologs to the query sequence and generating profile score. The amino acid residue substitution is then mapped to the known protein 3D structures and position-specific independent counts (PSIC) scores are calculated for each of the two amino acids. Finally, PSIC score difference is computed. A PSIC score difference more than or equal to 1.5 is considered to be damaging. Based on PSIC score difference, PolyPhen ranks nsSNP into one of the following three categories: (a) Benign (b) Possibly damaging and (c) Probably damaging. A total of thirty-one nsSNPs in protein transcripts (NP_000225.1) of gene LIG1 (NM_000234.1) were analysed for identification of deleterious variant(s).

### Identification of nonsynonymous single nucleotide polymorphism influencing protein stability

Cologne University Protein Stability Analysis Tool (CUPSAT) (Parthiban et al. 2006, 2007a, b) available at http://cupsat.tu-bs.de/ was applied to analyse changes in protein stability upon point mutation. The computational method makes use of amino acid-atom potentials and torsion angle distribution to assess amino acid environment of the mutation site (Parthiban et al. 2007a, b). The overall stability is calculated from atom and torsion angle potentials. In case of unfavourable torsion angles, atom potentials may have higher impact on stability which results in stabilising mutation (Parthiban et al. 2007). The output comprises of information about mutational site, its structural features, and information regarding changes in protein stability for 19 possible substitutions at the give position. The structure of gene product LIG1 was acquired from Protein Data Bank (PDB) (Berman et al. 2000), having PDB id 1x9n (A chain). The protein structure, native amino acid residue and its position was given as an input to the tool. A total of sixteen nsSNPs were evaluated for their influence on protein stability.

### Identification of single nucleotide polymorphism in splicing modifier binding site

FASTSNP (Yuan et al. 2006) a web-based tool, available at http://FASTSNP.ibms.sinica.edu.tw was used to determine polymorphism(s) in coding (nsSNP and sSNP) and in UTR regions of gene LIG1 influencing splicing regulation. FASTSNP is based on a decision tree principle and uses three web services: (i) ESEfinder (Cartegni et al. 2003; Smith et al. 2006) (ii) ESE-RESCUE (Fairbrother et al. 2002), and (iii) FAS-ESS (Wang et al. 2004) to predict impact of SNPs present in splicing modifier binding sites. SNPs present in Exonic Splicing Enhancer (ESE) sites are identified by ESEfinder and ESE-RESCUE tools. ESEfinder aids in identification of sSNPs located in ESE sites that will potentially weaken the binding site and ESE-RESCUE provides cross reference to the results from ESEfinder. While SNPs present in Exonic Splicing Silencer (ESS) site are identified by FAS-ESS tool. It also aids in identification of coding SNPs that will potentially abolish ESS sites. FASTSNP also computes a score based on the level of risk i.e., 0, 1, 2, 3, 4 and 5 indicating No, Very Low, Low, Medium, High and Very High risk.

### Identification of single nucleotide polymorphism in transcription factor binding site and in micro-RNA binding site

The authors used rSNP module from MAPPER web server available at http://genome.ufl.edu/mapper/mapper-main to identify SNPs present in binding site of one or more transcription factors in gene LIG1. The tool identifies TFBS in multiple genomes, by combining TRANSFAC (Matys et al. 2003, 2006) and JASPAR (Sandelin et al. 2004; Bryne et al. 2008; Portales-Casamar et al. 2010) data with profile hidden Markov model (HMMs) (Marinescu et al. 2005a, b) The gene LIG1 was given as an input to rSNP module and models from all available three libraries i.e., TRANSFAC matrices, TRANSFAC factors and JASPAR matrices were selected. The result comprises of a list of SNPs in TFBSs along with computed scores, these scores indicate changes in binding affinity of transcription factors. Furthermore, the tool does not limit its prediction to 5′UTR and promoter region but also extends it to intron region (Jun and Jing 2010).

Database of all miRNA binding sites within 200 nucleotides of a SNP (dbSMR) which may influence binding of miRNA, available at http://miracle.igib.res.in/polyreg/ was used to detect these SNPs (Hariharan et al. 2009). Both options present in database i.e., polymorphisms around predicted miRNA binding sites and polymorphisms around validated miRNA binding sites, were executed to identify SNPs influencing binding of miRNA to its target sites in gene LIG1.

### Modelling nsSNPs on protein structure and determining alterations in energy and RMSD

The structure of the gene product LIG1 was acquired from PDB, having PDB id 1x9n (A chain). The Swiss-PDB Viewer (Kaplan and Littlejohn 2001) was used for mapping mutations on structure. Selenomethionine residues present in the protein structures (native and mutant) were modified as Methionine using protein preparation wizard, Schrodinger, maestro (Schrodinger Inc. USA). The native and mutated structures were parameterized with amber03 force field and energy minimization was performed using GROMACS (Hess et al. 2008) (version 4.5.1) employing steepest descent algorithm. The RMSD values were computed using structural superimposition program from the Schrodinger suite. A total of seven nsSNPs were mapped onto the protein structure and analysed for change in energy and RMSD values from native structure.

## Results

### Dataset

The SNPs related to LIG1 gene were acquired from dbSNP database, build 132 (Sherry et al. 2001). Out of 792 SNPs, 52 (6.56%) SNPs were present in coding region of the gene (31 nsSNP and 21 sSNP), 3 SNPs (0.37%) in 3′UTR, 736 SNPs (92.92%) in intron region, 1 SNP (0.12%) in 5′UTR.

### Deleterious nonsynonymous single nucleotide polymorphisms predicted by SIFT server

Eleven nsSNPs were predicted to be deleterious with a tolerance index below 0.05. Lower the tolerance index or SIFT score, greater functional consequence an amino acid residue substitution is expected to have (Ng and Henikoff 2001). Four nsSNPs (rs111507847, rs3730947, rs34087182, rs11666150) had a tolerance index of 0.00, four nsSNPs (rs113944619, rs55686525, rs117019444, rs55950593) had a tolerance index of 0.01, two nsSNPs (rs3730863, rs3731003) had a tolerance index of 0.02, and the remaining one nsSNP (rs4987181) in the deleterious category had a tolerance index of 0.03. Seven nsSNPs (rs113944619, rs4987181, rs3730863, rs3730947, rs117019444, rs3731003, rs11666150) out of eleven nsSNPs predicted to be deleterious had a validated status (Table 1).

**Table 1:** Evaluation of nsSNPs from SIFT and PolyPhen servers

S. no.	dbSNP id	Allele	A.A. subs.	SIFT score	PSIC	Heterozygosity	Validation^a^
1	rs79652062	C/A	A17E	0.67	0.872	0.022	1, 6
2	rs3730855	C/T	A24V	0.59	0.094	0.007	1, 2
3	rs41555118	C/T	S47F	0.11	1.662	N.D.	1
4	rs113944619	C/G	S51C	**0.01**	**1.548**	N.D.	1
5	rs4987181	C/T	P52L	**0.03**	**2.550**	0.005	1
6	rs3730863	C/T	R62W	**0.02**	**1.868**	0.003	1
7	rs4987070	A/G	D72G	0.33	**1.700**	N.D.	1
8	rs79897727	T/C	S91P	0.33	**1.548**	0.146	1, 2, 6
9	rs41549918	G/A	R94H	0.10	0.000	N.D.	1
10	rs12981963	C/T	P119L	0.07	**2.550**	0.009	1, 2
11	rs11879148	C/T	P203L	0.15	**2.550**	N.D.	1, 5
12	rs55686525	C/T	R235W	**0.01**	**2.257**	N.D.	
13	rs3730911	G/A	G249E	1.00	0.975	0.007	1, 2
14	rs3730933	A/G	N267S	0.42	1.441	0.017	1, 2, 6
15	rs111846131	A/T	Y289F	0.19	0.353	N.D.	
16	rs111507847	C/T	S318F	**0.00**	**1.729**	N.D.	
17	rs3730947	G/A	V349M	**0.00**	0.060	0.459	1, 2
18	rs117019444	C/T	L355F	**0.01**	0.990	N.D.	6
19	rs3730966	G/A	V369I	1.00	0.501	0.004	1
20	rs4987068	G/A	R409H	0.25	0.345	0.024	1, 2, 6
21	rs3730980	A/G	M480I	1.00	0.840	0.259	1, 2
22	rs112555243	A/G	K487R	0.57	1.348	N.D.	
23	rs74929288	A/G	E497G	0.11	1.079	0.105	1, 2, 6
24	rs3731003	C/T	T614I	**0.02**	1.489	0.021	1, 2, 6
25	rs34087182	G/T	R641L	**0.00**	**2.840**	0.025	
26	rs56105837	G/A	D647N	0.49	0.286	N.D.	
27	rs55950593	C/T	R672C	**0.01**	**1.660**	N.D.	–
28	rs3731008	G/T	R677L	0.05	1.331	0.002	1, 6
29	rs11668325	G/A	S839N	0.57	1.331	N.D.	1, 5
30	rs11666150	G/T	Q892H	**0.00**	**2.307**	0.009	1, 2, 5
31	rs61752349	A/C	T918P	0.23	0.104	N.D.	

The nsSNPs predicted to be intolerant or damaging are highlighted as bold

^a^Validation status description: *1* validated by multiple, independent submissions to the refSNP cluster, *2* validated by frequency or genotype data: minor alleles observed in at least two chromosomes, *3* validated by submitter confirmation, *4* all alleles have been observed in at least two chromosomes a piece, *5* genotype by HapMap project, *6* SNP has been sequenced in 1,000 genome project

### Damaging nonsynonymous single nucleotide polymorphism predicted by PolyPhen

Thirteen nsSNPs out of thirty-one nsSNPs were predicted to be either possibly damaging or probably damaging and had PSIC score difference in the range of 1.548 and 2.840 (Table 1). Out of these thirteen nsSNPs, eight nsSNPs (rs113944619, rs4987181, rs12981963, rs11879148, rs55686525, rs111507847, rs34087182, and rs11666150) were put into the category of probably damaging and the remaining five nsSNPs (rs41555118, rs3730863, rs4987070, rs79897727, rs55950593) were put into the category of possibly damaging by the program. Eight nsSNPs (rs41555118, rs3730863, rs4987070, rs79897727, rs113944619, rs4987181, rs12981963, rs11879148) out of thirteen nsSNPs predicted to be in the category of either possibly damaging or probably damaging had validated status. It was observed that six nsSNPs (rs113944619, rs4987181, rs55686525, rs111507847, rs34087182, rs11666150) predicted to be probably damaging by PolyPhen server were also predicted deleterious by SIFT server. While two nsSNPs (rs3730863, rs55950593) predicted to be possibly damaging by PolyPhen server were also predicted to be deleterious by SIFT server. This shows a significant level of correlation between the results from evolutionary-based approach (SIFT) and structural-based approach (PolyPhen). The highly damaging nsSNP (rs34087182) had a PSIC score difference of 2.840 and SIFT score 0.00.

### Nonsynonymous single nucleotide polymorphism responsible for destabilising protein structure

CUPSAT identified two nsSNPs (rs3731003 and rs11666150) out of sixteen nsSNPs to be influencing over all stability of the protein structure. Ten nsSNPs (rs3730933, rs111846131, rs111507847, rs3730947, rs3730966, rs4987068, rs112555243, rs74929288, rs55950593, rs11668325) only exhibited unfavourable changes in torsion angles with no influence on overall stability of protein (Table 2). The nsSNP rs11666150 predicted to be destabilising protein structure was also predicted damaging by SIFT server (SIFT score 0.00) and PolyPhen server (PSIC score difference 2.307).

**Table 2:** Change in protein structure and DDG upon point mutation

S. no.	dbSNP id	A.A. subs.	Changes predicted in protein structure by CUPSAT Server				
			Overall stability	Torsion	Predicted change in DDG (kcal/mol)	Solvent accessibility (%)	Secondary str. element
1	rs3730933	N267S	Stabilising	Unfavourable (−140.5°, 79.5°)	0.95	38.93	Others (turn, coils, etc.)
2	rs111846131	Y289F	Stabilising	Unfavourable (−53.2°, −37.0°	3.48	0.47	Helix
3	rs111507847	S318F	Stabilising	Unfavourable (−71.5°, −40.2°)	0.35	0.0	Helix
4	rs3730947	V349M	Stabilising	Unfavourable(−71.6°, 145.6°)	1.6	12.36	Others (turn, coils, etc.)
5	rs117019444	L355F	Stabilising	Favourable (−49.5°, −58.8°)	0.66	6.01	Helix
6	rs3730966	V369I	Stabilising	Unfavourable (−52.7°, −60.2°)	2.55	0.0	Helix
7	rs4987068	R409H	Stabilising	Unfavourable (−58.2°, −22.3°**)**	1.42	20.09	Helix
8	rs112555243	K487R	Stabilising	Unfavourable (−55.7°, 136.2°)	1.01	41.81	Others (turn, coils, etc.)
9	rs74929288	E497G	Stabilising	Unfavourable (−57.2°, −33.5°)	1.54	66.97	Helix
10	rs3731003	T614I	Destabilising	Unfavourable (−88.3°, −20.1°)	−0.07	45.42	Others (turn, coils, etc.)
11	rs34087182	R641L	Stabilising	Favourable (−74.1°, 142.9°)	1.05	11.79	Others (turn, coils, etc.)
12	rs56105837	D647N	Stabilising	Favourable (−70.8°, 76.6°)	1.52	38.17	Others (turn, coils, etc.)
13	rs55950593	R672C	Stabilising	Unfavourable (−89.6°, 22.7°)	0.66	62.45	Others (turn, coils, etc.)
14	rs3731008	R677L	Stabilising	Favourable (−51.0°, −48.2°)	0.85	47.16	Helix
15	rs11668325	S839 N	Stabilising	Unfavourable (−158.4°, −41.3°)	3.05	49.49	Others (turn, coils, etc.)
16	rs11666150	Q892H	Destabilising	Unfavourable (−62.8°, −47.0°)	−0.07	54.87	Helix

### Functional single nucleotide polymorphism in splicing modifiers binding site

FASTSNP predicted twenty SNPs to be influencing splicing regulation by their presence in splicing modifiers (enhancers and silencers) binding site (Table 3) (krawczak et al. 2000). Sixteen SNPs predicted to be influencing splicing regulation had a risk in range of 2–3 (low to medium) and remaining four SNPs with a risk in range of 3–4 (medium to high). Interestingly, two SNPs rs20581 and rs20580 were also highlighted in recent studies for their functional importance (Chang et al. 2008; Lee et al. 2008; Liu et al. 2009). None of the SNPs in UTR were reported to be present in splicing modifier binding sites.

**Table 3:** SNPs present in splicing modifier binding sites

S. no.	dbSNP id	Possible functional effect	Risk
1	rs41546017	Sense/synonymous; splicing regulation	2–3
2	rs35100567	Sense/synonymous; splicing regulation	2–3
3	rs1126814	Missense (conservative); splicing regulation	2–3
4	rs20581	Sense/synonymous; splicing regulation	2–3
5	rs35485148	Sense/synonymous; splicing regulation	2–3
6	rs3731027	Sense/synonymous; splicing regulation	2–3
7	rs56165744	Sense/synonymous; splicing regulation	2–3
8	rs3731008	Missense (conservative); splicing regulation	2–3
9	rs55817698	Sense/synonymous; splicing regulation	2–3
10	rs3730933	Missense (conservative); splicing regulation	2–3
11	rs3730911	Missense (conservative); splicing regulation	2–3
12	rs55686525	Missense (conservative); splicing regulation	2–3
13	rs11879148	Missense (conservative); splicing regulation	2–3
14	rs20580	Sense/synonymous; splicing regulation	2–3
15	rs12981963	Missense (conservative); splicing regulation	2–3
16	rs3730855	Missense (conservative); splicing regulation	2–3
17	rs3731003	Missense (non-conservative); splicing regulation	3–4
18	rs4987070	Missense (non-conservative); splicing regulation	3–4
19	rs3730863	Missense (non-conservative); splicing regulation	3–4
20	rs4987181	Missense (non-conservative); splicing regulation	3–4

### Functional single nucleotide polymorphism in transcription factor binding site, micro RNA binding site, and in promoter region

Gene LIG1 contains binding sites for a number of transcription factors which may mediate increased expression in dormant cells in response to growth factors (Noguiez et al. 1992). The presence of transcription factor binding site is not limited to 5′UTR or to promoter region but it also extends to intronic region (Jun and Jing 2010). Nine SNPs were predicted to be present in transcription factor binding site. Five SNPs (rs3730842, rs75696040, rs74747924, rs7246696 and rs3730840) in intron and four SNPs (rs3730838, rs752084, rs3730836 and rs79501686) in promoter region were predicted to be present in TFBS. Two SNPs (rs75696040 and rs74747924) were predicted to be present in the binding site of MZF1 transcription factor in chromosomal region between 48,673,165 to 48,673,177 on chromosome 19. Other than SNP rs79501686, all SNPs gave a score difference of more than 2, indicating the presence of SNP substantially influences binding affinity of transcription factors (Table 4). None of the SNPs by dbSMR were reported to be influencing binding of micro RNA in gene LIG1.

**Table 4:** SNPs present in transcription factor binding site

dbSNP id	Region	Score^a^ (Bef./Aft/Diff)	Model	Factor	Strand	chr	Chr. st.	Chr. end
rs3730842	Intron	−/3.6/**3.6**	T00601	NF-1 (-like proteins)	+	19	48,672,996	48,673,009
rs75696040	Intron	−/3.6/**3.6**	M00084	MZF1	–	19	48,673,165	48,673,177
rs74747924	Intron	−/3.6/**3.6**	M00084	MZF1	–	19	48,673,165	48,673,177
rs7246696	Intron	−/3.6/**3.6**	T03717	ZAP1	–	19	48,673,210	48,673,220
rs3730840	Intron	−/2.7/**2.7**	M00720	CAC-binding protein	–	19	48,673,455	48,673,463
rs3730838	Promoter	1.2/5.2/**4.0**	M00442	ABF	+	19	48,673,851	48,673,866
rs752084	Promoter	−/3.1/**3.1**	M00273	R	+	19	48,673,926	48,673,944
rs3730836	Promoter	−/2.3/**2.3**	MA0054	myb.Ph3	+	19	48,674,356	48,674,364
rs79501686	Promoter	0.1/1.5/1.4	MA0073	RREB1	–	19	48,674,851	48,674,870

^a^Description of Scores, *Bef* score of the TFBS in absence of SNP, *Aft* score of the TFBS in presence of SNP, *Diff* difference in Bef and Aft scores

The scores in bold indicate substantial change in binding affinity of transcription factors in presence of SNP

### Mapping and analysis of mutants on protein structure

**Fig. 1:**
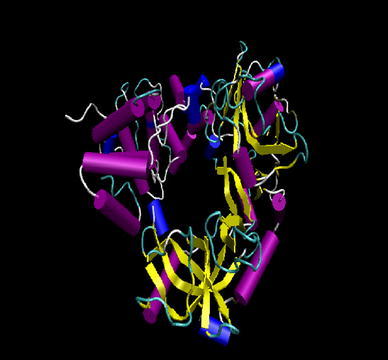
Native structure of protein DNA Ligase I

**Fig. 2:**
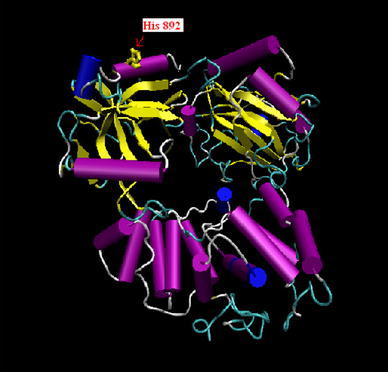
Mutant model of mapped nsSNP (rs11666150) on the structure of protein DNA Ligase I

Seven nsSNPs (rs11666150, rs55950593, rs34087182, rs3731003, rs117019444, rs3730947 and rs111507847) predicted to be deleterious by SIFT or PolyPhen server and present between the residue number 262 and 901 were mapped on the protein structure (PDB id: 1x9n, A chain) of gene product LIG1. The amino acid residue substitution was performed using Swiss-PDB Viewer to get seven mutant modelled protein structures for SNPs rs117019444, rs111507847, rs3730947, rs3731003, rs34087182, rs55950593, and rs11666150. The total energy of the native structure (1x9n, A chain, Fig. 1) and the seven mutant modelled protein structures for SNPs rs117019444, rs111507847, rs3730947, rs3731003, rs34087182, rs55950593, and rs11666150 was −52745.32, −62163.56, −40160.3672, −59187.7773, −57279.74, −59290.78, −53570.56 and −41863.19 kJ/mol, respectively (Table 5). It can be observed from Table 5 that the RMSD values fall in range of 0.00522673–0.0361993 and do not suggest much deviation while significant changes in energy of mutant structures can be observed. The mutant protein models for SNPs rs11666150 (Fig. 2) and rs111507847 showed an increase in energy compared to the energy of native structure. The result for nsSNP rs11666150 correlates with results given by SIFT, PolyPhen and CUPSAT servers. The native and mutant protein molecule structures were visualised using Visual Molecular Dynamics (VMD) program (Humphrey et al. 1996).

**Table 5:** RMSD value and overall energy of mutant protein structures

dbSNP id	A.A. subs.	RMSD (Å)	Energy (KJ/mol)
rs117019444	S318F	0.0242222	−62163.56
rs111507847	V349M	0.0129564	−40160.3672
rs3730947	L355F	0.00522673	−59187.7773
rs3731003	T614I	0.0246375	−57279.74
rs34087182	R641L	0.0361993	−59290.78
rs55950593	R672C	0.0222391	−53570.56
rs11666150	Q892H	0.0196504	−41863.19

## Discussion

Laboratory-based techniques are most accurate and conclusive in distinguishing functional SNPs from non-functional SNPs (Chen and Sullivan 2003). But large number of SNPs present in human genome makes execution of laboratory techniques very demanding in terms of time, cost, and labour. On contrary, *in silico* methods can help in distinguishing potentially functional SNPs from neutral SNPs present in a gene.

**Fig. 3:**
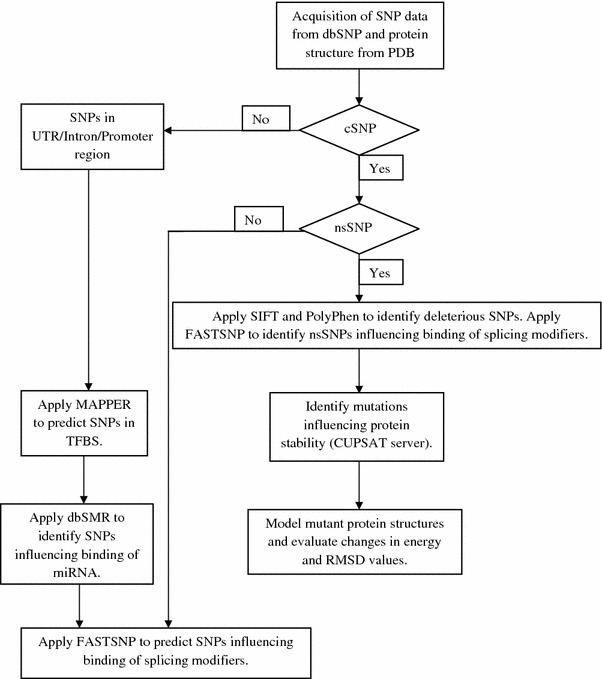
Workflow along with the tools and databases used to identify potential functional SNPs in human gene LIG1

The computational pipeline (Fig. 3) was applied to all SNPs linked to gene LIG1as cited in dbSNP. Eleven and thirteen nsSNPs were predicted to be deleterious by SIFT and PolyPhen server, respectively. Eight nsSNPs were predicted to be deleterious by both SIFT and PolyPhen server. Evaluation of protein stability upon point mutation by CUPSAT server showed two nsSNPs (rs11666150 and rs3731003) to be able to destabilize protein structure. Out of seven mutant models of nsSNPs only two nsSNPs (rs11666150 and rs111507847) mutant models demonstrated significant change in energy compared to native structure of protein. Interestingly, one nsSNP (rs11666150) was predicted to be intolerant, probably damaging and destabilizing by SIFT, PolyPhen and CUPSAT servers, respectively, and also its mutant structure showed a significant change in energy level. FASTSNP web server predicted twenty SNPs to be influencing splicing regulation and four were predicted with a risk in range of 3–4 (medium to high). Nine SNPs from intron and promoter region were predicted by rSNP module from MAPPER to be influencing binding of transcription factor. The *in silico* study was well-focussed on SNPs present in all regions of gene LIG1 as regulatory region SNPs may also be disease causatives (Hudson 2003; Yan et al. 2002). Furthermore, results of the study were in concordance with the results from recent studies (Chang et al. 2008; Lee et al. 2008; Liu et al. 2009; Ryu et al. 2009).

A large variety of tools are freely available for identification of potentially functional SNPs in a gene and each tool has different perspective for same biological problem (Thusberg and Vihinen 2009). The choice of computational tools to be used in an analysis is made on the nature of functional SNP to be identified and the amount of data and information being available for a given gene.

## Conclusion

In this study nsSNP rs11666150 was found damaging by all the functional nsSNP prediction servers used. Further, its mutant structure demonstrated significant overall energy change as compared to the native structure. In this analysis, SNPs influencing binding of transcription factor and splicing modifier binding site are also predicted. However, studies will be required for in vitro validation of potentially functional SNPs in LIG1 and eventually will lead to development of better drugs against DNA ligase I deficiency (MIM: 126391). The authors suppose that the computational pipeline used in this study may also apply to any other human gene to identify potentially functional SNPs in it.
